# Trace element concentrations in blood samples from dairy cows with uterine torsion and their neonatal calves

**DOI:** 10.14202/vetworld.2023.2533-2537

**Published:** 2023-12-28

**Authors:** Kei Kazama, Kazutoshi Sugita, Ken Onda

**Affiliations:** Department of Veterinary Medicine, Azabu University, School of Veterinary Medicine, 17-71 Fuchinobe 1-chome, Chuo-ku, Sagamihara 252-5201, Japan

**Keywords:** cattle, dystocia, mortality calves, neonate, trace elements

## Abstract

**Background and Aim:**

Mineral deficiencies can lead to dystocia and abnormalities in neonates. Stillbirth of neonatal calves in dairy cows due to dystocia has become an economic problem. Uterine torsion (UT) is a common form of dystocia observed in dairy cows. However, to the best of our knowledge, there have been no reports on the characteristics of serum trace element concentrations in dairy cows with UT. This study aimed to comprehensively measure serum trace element concentrations in dairy cows with UT and dystocia.

**Materials and Methods:**

Dairy cows with (n = 15) and without (n = 27) UT and neonates (n = 9 and n = 26, respectively) were included in this study. Blood samples (10 mL) were collected, and serum trace element concentrations were evaluated using inductively coupled plasma mass spectrophotometry.

**Results:**

The mortality rate at birth was significantly higher in calves delivered by cows with UT than those delivered by cows without UT. The odds ratio for mortality rate at birth in dairy cows with UT was 7.85. Serum zinc (Zn) levels were significantly lower in cows with UT than in cows without UT (p = 0.01). The copper: Zn ratio was significantly higher in cows with UT than in cows without torsion (p = 0.05). In contrast, serum Cobalt (Co) concentrations were significantly higher in neonates from cows with UT than in neonates from cows without UT (p = 0.01).

**Conclusion:**

Uterine torsion is associated with a high mortality rate at birth and considerable economic losses. Cows with UT had low serum Zn levels, and neonates born to these cows had high serum Co levels.

## Introduction

There is a high incidence of dystocia during parturition in cows with hypocalcemia and hypophosphatemia [1]. In addition, cows with hypocalcemia during parturition have a higher incidence of stillbirth [1]. Dystocia is an abnormal difficulty in birthing that can be observed in dairy cows during calving.

Uterine torsion (UT) is a common cause of dystocia in dairy cows [2, 3] and can result in local ischemia, fetal death, or even death of the cow [2]. Notably, calcium administration by veterinarians is higher in cows with UT than in cows with other forms of dystocia [4]. It has also been reported that dystocia cows have lower plasma zinc (Zn) levels than eutocia cows [5].

Serum levels of copper (Cu) and Zn are lower in women with spontaneous abortion [6]. In addition, lower serum Zn levels have been observed in women with preterm delivery or miscarriage compared with controls [7]. Low Zn and decreased Fe levels are associated with abortion [8]. Abnormal levels of Cu and Zn in the blood are associated with diseases, and the homeostasis of these minerals is important for maintaining health. Abnormal Cu/Zn ratio has been reported to be related to spontaneous abortion [6]. Cobalt (Co) is an important element required for fetal growth, and low levels of Co are associated with an increased risk of macrosomia [9]. Serum selenium (Se) concentrations are lower in premature infants than mature infants, and maternal serum Se concentrations correlate positively with neonatal birth weight [10]. In addition, deficiency of various minerals may lead to dystocia and neonatal abnormalities. However, to the best of our knowledge, there have been no reports on the characteristics of serum trace element concentrations in UT dairy cows.

The aim of this study was to comprehensively measure serum trace element concentrations in dairy cows with UT and dystocia.

## Materials and Methods

### Ethical approval

This study was approved by the Animal Care and Use Committee of Azabu University School of Veterinary Medicine (approval number: 171113-1).

### Study period and location

The study was conducted from July 2019 to May 2021 at Azabu University, Kanagawa prefecture, Japan, using the samples received from Hokkaido Higashi Agricultural Mutual Aid Association, Hokkaido, Japan, from May 2017 to March 2019.

### Samples

Forty-two Holstein cows bred in the Kushiro area of Hokkaido, Japan, were included in this study. Maternal cows with UT (MUT, n = 15), maternal cows without UT (non-MUT, n = 27), neonatal calves from MUT (NUT, n = 9), and neonatal calves from non-MUT (non-NUT, n = 26) were divided into groups based on their condition at the time of delivery. Jugular blood samples were collected from the cows immediately after birth and the neonatal calves before colostrum feeding. Table-1 shows the calving number, gestational age, and mortality rate at birth in each group. Specimens could not be collected from some of the calves because they were stillborn. Blood samples were placed in plain tubes (Terumo Corporation, Tokyo, Japan) and incubated at 37°C for 15 min to allow for coagulation. Subsequently, the tubes were centrifuged at 1,700× g for 15 min at 4°C, and the serum was separated and stored at −20°C until use.

**Table-1:** Calving number, gestational age, and mortality rate at birth of cows with uterine torsion (MUT) and without uterine torsion (non-MUT).

Groups	Calving number	Gestational age (days)	Mortality rate at birth
non-MUT	3.2 ± 1.7	281.14 ± 4.94	2/27
MUT	3.5 ± 2.3	280.53 ± 10.24	6/15
Odds ratio			7.85*

* p < 0.05

### Serum mineral analysis

Serum samples (60 μL) were added to nitric acid (1 mL: 63% wt/wt) in tetrafluoro methoxyl inserts and then digested at 50°C for 2 min, 30°C for 1 min, and 180°C for 20 min using a microwave digestion system (START D, Milestone General K.K., Kanagawa, Japan). Digested samples were diluted 250-fold in a decomposition matrix before undergoing inductively coupled plasma mass spectrophotometry (ICP-MS) analysis. Nitric acid (1%, v/v) in distilled deionized water was used as the decomposition matrix.

### Instruments

The trace elements in each sample were determined using an ICP-MS instrument (Agilent 7700×, Agilent Technologies Co., Tokyo, Japan), equipped with a quadrupole mass spectrometer and an octapole reaction cell. The ICP-MS instrument operating conditions are summarized in Table-S1. To account for matrix effects caused by major elements, internal standard correction was performed for the ICP-MS measurements using tellurium and iridium as internal standard elements (Merck, Tokyo, Japan). Purified water (18.2 MQ cm) was prepared using a Milli Q SP-TOC system (Nihon Millipore Kogyo, Tokyo, Japan) according to the manufacturer’s instructions.

**Table-S1:** Operating conditions for the ICP-MS instrument.

ICP-MS: Agilent 7700×	Conditions
Plasma conditions:	
RF power	1.55 kW
Plasma gas flow rate	15.0 L/min Ar
Auxiliary gas flow rate	0.90 L/min Ar
Makeup gas flow rate	0 L/min Ar
Carrier gas flow rate	1.0 L/min Ar
Sampling depth (mm from load coil)	He mode: 8.00 mm H_2_ mode: 10.00 mm
Cell gas	He mode: 4.3 mL/min Ar
	H_2_ mode: 6.0 mL/min Ar
Nebulizer	Micro Mist
Sample uptake rate	0.45 mL/min
Data acquisition:	
Accumulation time	0.3–1.0 s/point
Data point	3 points/peak
Repetition	3 times

ICP-MS=Inductively coupled plasma mass spectrophotometry

### Chemicals

The standard solutions for creating the calibration curves in the ICP-MS measurements were prepared by diluting commercial multi-element standard stock solutions (TraceCERT®, Sigma-Aldrich, Tokyo, Japan) and a Molybdenum ICP-MS Standard (Wako Pure Chemical Industries Inc., Osaka, Japan). The nitric acid solution used was of industry-grade (Kanto Chemical Co., Tokyo, Japan).

### Statistical analysis

Data are expressed as the mean ± standard deviation. As verified by the Shapiro–Wilk test, the data obtained did not have a normal distribution. We compared serum trace element levels between the UT and non-UT groups using the Mann–Whitney U-test. All statistical analyses were performed using EZR (Saitama Medical Center, Jichi Medical University, Saitama, Japan), which is a graphical user interface for R (The R Foundation for Statistical Computing, Vienna, Austria). EZR is a modified version of the R commander, designed to add statistical functions frequently used in biostatistics. p < 0.05 was considered statistically significant, whereas p < 0.10 was considered as a significant tendency.

## Results

There were no significant differences in calving number or gestational age. Calves delivered by cows with UT had a significantly higher mortality rate at birth (odds ratio = 7.85) than those delivered by cows without UT (Table-1).

Table-2 shows the concentrations of maternal cow serum trace elements. Serum Zn concentrations were significantly lower in the MUT group than in the non-MUT group (p < 0.01). Serum Se concentrations were higher in the MUT group than in the non-MUT group (p = 0.0673). Moreover, the Cu/Zn ratio was significantly higher in the MUT group than that in the non-MUT group (p < 0.05).

**Table-2:** Serum trace element concentrations of maternal cows.

Trace elements	MUT (μg/dL)	N	non-MUT (μg/dL)	n	p- value
Cr	1.92 ± 1.36	9	2.09 ± 1.74	16	NS
Mn	0.60 ± 0.60	5	1.18 ± 0.98	16	NS
^56^Fe	123.21 ± 49.70	9	157.70 ± 101.10	19	NS
^57^Fe	120.68 ± 44.95	9	154.65 ± 100.98	19	NS
Co	0.12 ± 0.05	7	0.19 ± 0.11	14	NS
Ga	1.42 ± 0.64	9	1.34 ± 0.69	18	NS
Se	4.53 ± 1.64	9	3.62 ± 1.71	18	0.067
Rb	18.08 ± 3.58	9	16.74 ± 6.19	19	NS
Sr	5.66 ± 1.28	9	7.14 ± 4.72	18	NS
Mo	2.86 ± 1.84	9	3.68 ± 4.29	19	NS
Cs	0.17 ± 0.05	9	0.19 ± 0.10	18	NS
Ba	9.21 ± 4.95	9	8.74 ± 3.87	18	NS
Cu	69.83 ± 21.67	9	82.33 ± 44.37	19	NS
Zn	49.84 ± 7.95	5	112.94 ± 61.60	15	0.008
Cu/Zn ratios	1.60 ± 0.45	5	1.10 ± 1.25	15	0.025

NS=Non significant

The neonatal serum trace element concentrations are shown in Table-3. The serum Co concentrations in the NUT group were significantly higher than those in the non-NUT group (p < 0.01). Furthermore, the NUT group had lower Zn concentrations (p = 0.0583) and higher Se concentrations (p = 0.0726) compared to the non-NUT.

**Table-3:** Serum trace element concentrations of neonatal calves.

Trace elements	NUT (μg/dL)	N	non-NUT (μg/dL)	n	p- value
Cr	1.59 ± 1.21	5	1.35 ± 1.38	14	NS
Mn	0.49 ± 0.20	2	1.25 ± 1.26	16	NS
^56^Fe	147.43 ± 47.47	6	185.33 ± 96.29	20	NS
^57^Fe	138.40 ± 44.52	6	187.83 ± 96.76	20	NS
Co	0.54 ± 0.23	4	0.17 ± 0.12	14	0.005
Ga	1.14 ± 0.24	5	1.36 ± 0.85	18	NS
Se	3.33 ± 0.68	6	2.36 ± 1.26	20	0.073
Rb	23.44 ± 3.01	6	22.28 ± 8.27	20	NS
Sr	4.72 ± 0.89	6	6.57 ± 4.87	19	NS
Mo	1.87 ± 1.22	6	2.74 ± 3.14	20	NS
Cs	0.23 ± 0.08	6	0.23 ± 0.10	19	NS
Ba	6.70 ± 2.70	6	8.76 ± 5.27	19	NS
Cu	19.14 ± 4.22	5	23.37 ± 7.04	19	NS
Zn	87.75 ± 62.34	5	182.61 ± 105.18	17	0.058
Cu/Zn ratios	0.40 ± 0.38	5	0.18 ± 0.14	17	NS

NS=Non significant

## Discussion

Uterine torsion can result in local ischemia, leading to fetal death, potentially resulting in the death of the cow [2]. In calves born to cows with UT, the mortality rate at birth was 7.85 times higher than that in calves born without torsion. Therefore, UT is considered to cause great economic losses associated with the loss of calves.

Zinc is an essential component of various enzymes, including DNA and RNA synthesis [11]. Zinc deficiency decreases immunity and resistance to infectious disease in humans and animals [12]. Serum Zn concentrations in dairy cows have been shown to decrease in cases of subclinical ketosis [13]. Serum Zn concentrations also decreased in dairy cows with subclinical hypocalcemia [14]. Zinc deficiency is suspected to contribute to immunosuppression, decreased productivity, and poor reproductive performance. Maternal serum Zn concentrations were significantly lower in cows with UT than in those without torsion. Blood Zn concentrations also decreased before, during, and 1 week after parturition [14, 15]. Similarly, the results of this study showed a marked decrease in serum Zn concentration in cows with UT, whereas a decreased periparturient Zn concentration in healthy cows. Copper/Zn ratios were significantly higher in cows with UT than in those without torsion as serum Zn concentration decreased. Copper/Zn ratios have been reported to be associated with malnutrition, increased oxidative stress, inflammation, and a disrupted immune status [16]. The Cu/Zn ratio is considered a clinically useful index compared to the concentration of Cu or Zn alone [17]. The Cu/Zn ratio has been reported to be a useful diagnostic marker for ectopic pregnancy related to oxidative stress in humans [18]. Uterine torsion induces significant oxidative stress at the tissue level due to the increased production of free radicals, as indicated by the high levels of malondialdehyde, superoxide dismutase, and glutathione [19]. Therefore, Cu/Zn ratio may provide valuable insights into the pathology of UT in dairy cows.

Selenium is a trace element that plays an important role in animal health and performance [20]. In cattle, Se deficiency can contribute to retained placenta [21], mastitis [22], reduced fertility, and metritis [23], all of which can have a significant economic impact in terms of reduction in milk production and poor reproductive performance. However, a study on pregnant cows fed with Se demonstrated that subclinical Se toxicosis could lead to stillbirth, which may adversely affect both pregnancy outcomes and the bovine immune system [24]. In the present study, serum Se concentrations tended to be higher in both cows with UT and in calves born to cows with UT, although the difference was not statistically significant. However, the association between Se and UT in dairy cows remains unclear and requires further investigation.

Cobalt is essential for the formation of vitamin B12 [25]. Moreover, vitamin B12 deficiency is known to cause macrocytosis [26]. Buffaloes with UT have been reported to show macrocytosis [27]. However, neonatal calves born to cows with UT had significantly higher serum Co concentrations (p < 0.01). Cobalt chloride has traditionally been used to treat anemia in pregnant women, infants, and patients with chronic anemia undergoing long-term hemodialysis. Cobalt chloride induces *in vivo* hypoxia-like reactions, including erythropoiesis and angiogenesis [28]. Uterine torsion leads to local ischemia [2], resulting in an insufficient supply of oxygen to the fetus and leading to potential hypoxemia. Hypoxia-inducible factor (HIF) activates the expression of genes that contain hypoxia response element and helps cells adapt to hypoxia [29]. Hypoxia-inducible factor is a heterodimeric transcription factor composed of oxygen-regulated alpha subunits (HIF −1α, −2α, and −3α) [30]. Cobalt and HIF −1α mRNA have been reported to correlate dose- and time-dependent [31]. Therefore, high serum Co levels in neonatal calves born to cows with UT may protect against ischemic hypoxemia. The mechanism underlying high Co levels in neonatal calves requires further investigation.

## Conclusion

Uterine torsion is associated with a high birth mortality rate and significant economic loss. Cows with UT have low serum Zn levels and a high Cu/Zn ratio, which may predict reduced disease resistance. Calves born to cows with UT have high levels of serum Co and can adapt to ischemic hypoxemia. To the best of our knowledge, this is the first study to examine trace mineral levels in the serum of neonatal calves and dairy cows with UT. Further investigation is needed to elucidate the relationship between UT and trace mineral levels.

## Data Availability

The datasets generated and/or analyzed during the present study are available from the corresponding author on a reasonable request.

## Authors’ Contributions

KK: Material preparation, data collection, sample analysis, and the first draft of the manuscript. KS: Analyzed the data. KO: Designed the study and revised the manuscript. KK, KS, and KO: Interpreted the data and critically revised the manuscript for important intellectual content. All authors have read, reviewed, and approved the final version of this manuscript.
